# Supplementary material for: Genetic Basis of Resistance to Warrior (-) Yellow Rust Race at the Seedling Stage in Current Central and Northern European Winter Wheat Germplasm
Source: Plants (Basel). 2023 Jan 17;12(3):420. doi: 10.3390/plants12030420 (PMC9920722; doi:10.3390/plants12030420)
Supplement: Supplementary file 1 [file plants-12-00420-s001.zip › Supplementary Figures_FS et al..docx]

*Warrior (-) FS 53/20, Replication 1*


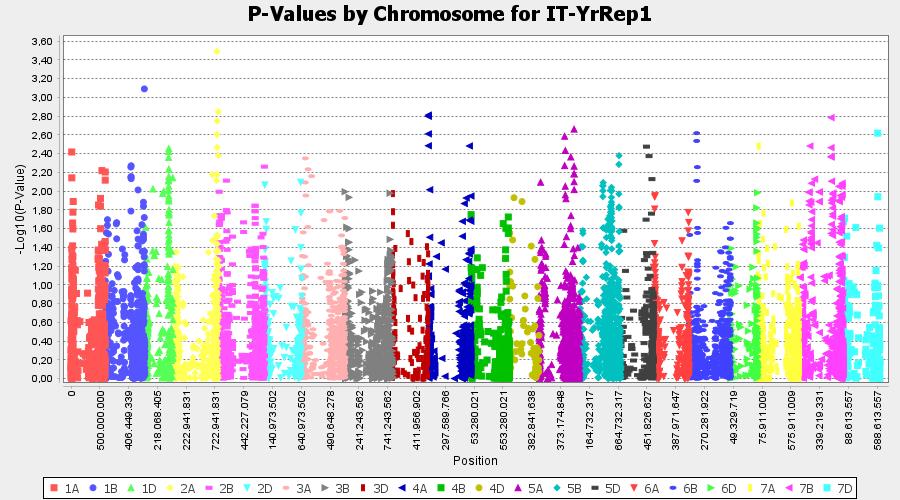


*Warrior (-) FS 53/20, Replication 2*


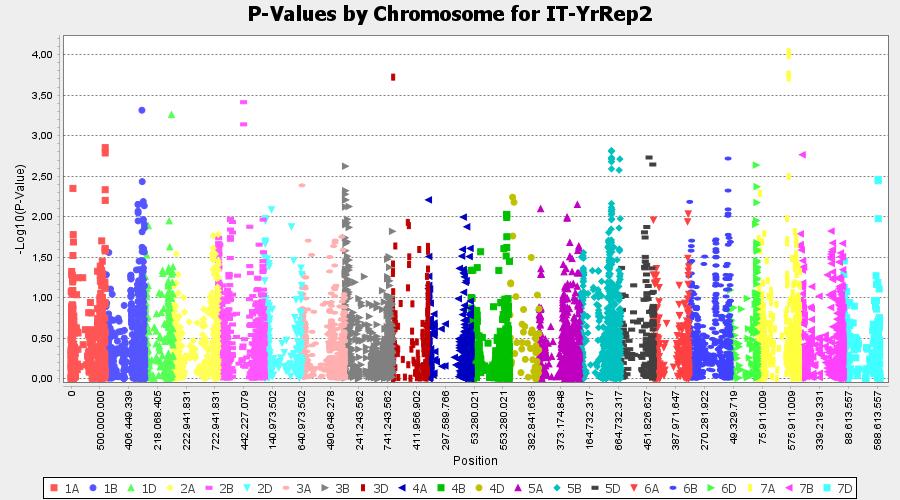


*Warrior (-) FS 53/20, Replication 3*

*
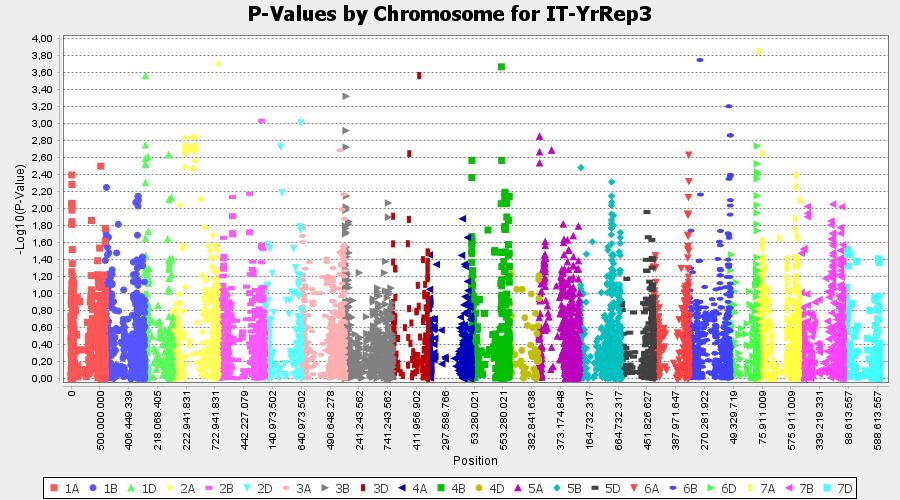
*

*Warrior (-) G 23/19, Replication 1*


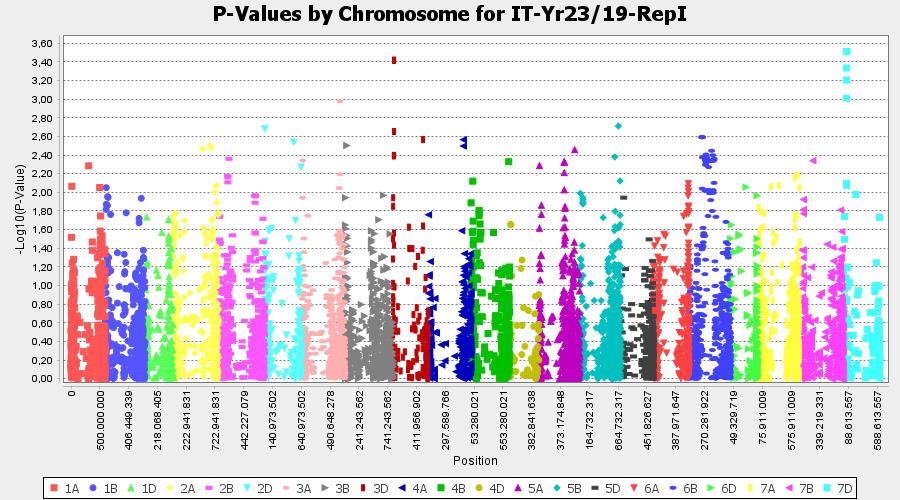


*Warrior (-) G 23/19, Replication 2*


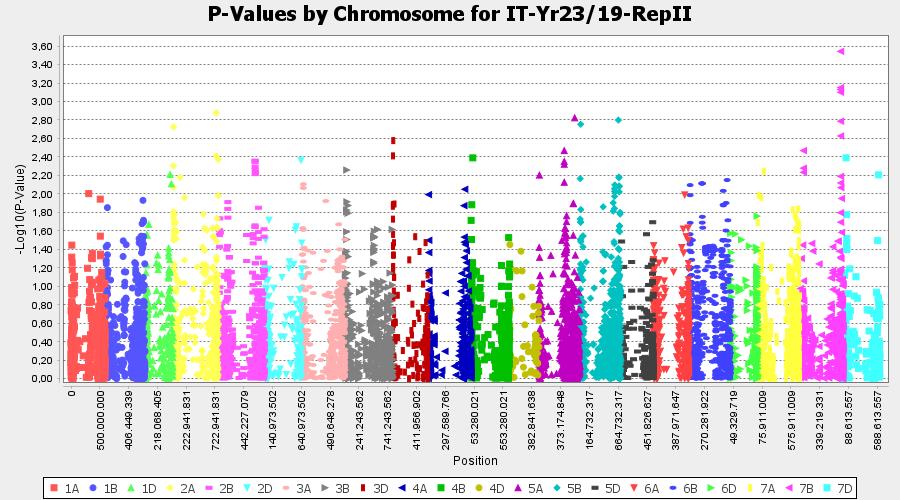


*Warrior (-) G 23/19, Replication 3*


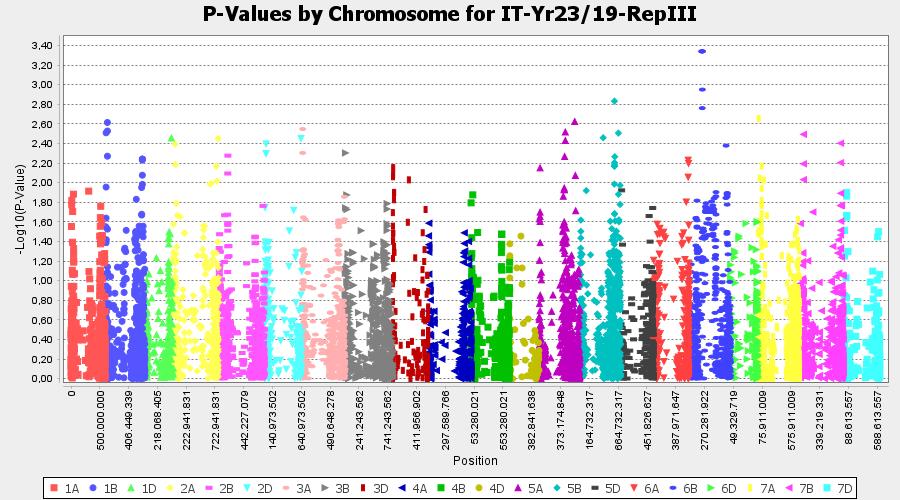


**Figure S1.** Manhattan plots showing the association of SNPs in the 229 genotypes used in the GWAS for yellow rust resistance at the seedling stage for three replications conducted for *Pst* pathotypes *Warrior (-) FS 53/20* and *Warrior (-) G 23/19*. SNPs with the -Log 10(P-value) > 3 in the Manhattan plots are significant and were listed in Table S2.
